# Drug Release from Lipid Microparticles—Insights into Drug Incorporation and the Influence of Physiological Factors

**DOI:** 10.3390/pharmaceutics16040545

**Published:** 2024-04-15

**Authors:** Eliza Wolska, Karolina Sadowska

**Affiliations:** 1 Department of Pharmaceutical Technology, Medical University of Gdansk, Hallera 107, 80-416 Gdansk, Poland; 2 Student Chapter of the International Society of Pharmaceutical Engineering (ISPE), Medical University of Gdansk, Hallera 107, 80-416 Gdansk, Poland

**Keywords:** release study, lysozyme enzyme, tear fluid, polysorbate, indomethacin, solid lipid microparticles

## Abstract

The aim of this study was to assess the impact of physiological factors, namely tear fluid and lysozyme enzyme, as well as surfactant polysorbate, on the release profile from solid lipid microparticles (SLM), in the form of dispersion intended for ocular application. Indomethacin (Ind) was used as a model drug substance and a release study was performed by applying the dialysis bag method. Conducting release studies taking into account physiological factors is expected to improve development and screening studies, as well as support the regulatory assessment of this multi-compartment lipid dosage form. The effect of the lysozyme was directly related to its effect on lipid microparticles, as it occurred only in their presence (no effect on the solubility of Ind). Polysorbate also turned out to be an important factor interacting with the SLM surface, which determined the release of Ind from SLM. However, in study models without tear fluid or lysozyme, the release of Ind did not exceed 60% within 96 h. Ultimately, only the simultaneous application of artificial tear fluid, lysozyme, and polysorbate allowed for the release of 100% of Ind through the SLM dispersion. The examination of the residues after the release studies indicated the possibility of releasing 100% of Ind from SLM without complete degradation of the microparticles’ matrix. The incubation of SLM with tear fluid confirmed a similar influence of physiological factors contained in tear fluid on the surface structure of SLM as that observed during the in vitro studies.

## 1. Introduction

Topical instillation of eye drops is still the preferred route of drug administration in ophthalmology [1]. However, the limitations, such as a short ocular contact time and ocular defense mechanisms (reflex blinking, tear turnover, nasolacrimal drainage, anatomical barriers), cause the bioavailability of the active substance administered in this way to be lower than 5% [1]. Therefore, efforts continue to search for and develop modern drug carriers that stay longer in the conjunctival sac and ensure a prolonged drug release. An example of such a carrier is the use of solid lipid microparticles (SLM), as a multi-compartment lipid-based formulation, providing a prolonged release effect. SLM dispersions are well-tolerated after ocular administration, according to in vivo studies on rabbits [2].

SLM are lipid particles that do not melt and instead remain solid at the human body temperature. SLM can create liquid (dispersion–suspension), semi-solid (concentrate), and solid (powder, implant, tablet) dosage forms. Therefore, they can be used in various routes of administration, not only topically to the eye but also to the skin or mucous membranes, orally, intramuscularly, or by inhalation [2,3,4,5,6,7,8,9,10]. The first generation of solid lipid particles were SLN (solid lipid nanoparticles). The composition of SLM is equivalent to that of SLN, but in the micrometer size range [11]. An important advantage of both SLM and SLN as drug carriers is their biodegradability. Furthermore, the advantages of SLM compared to SLN are the simpler production process and superior extended release properties due to their increased size and greater loading capability. Moreover, due to their micron dimensions, they do not raise toxicological concerns related to nanoparticles [12]. At the same time, SLM as drug carriers are significantly less studied and recognized than SLN.

Since lipid particles are a multi-compartment dosage form, the drug substance can be localized not only in the lipid matrix but also on the particle’s surface. Models of a homogeneous matrix, drug-enriched outer shell, and drug-enriched core have already been described [13,14]. A prolonged release effect is determined by a drug fraction localized in a solid lipid matrix. Despite many predictive models theoretically considering the method of binding and the exact location of the active substance in the lipid particles [13,15,16,17], it is extremely difficult to investigate thoroughly and thus understand this feature of the dosage form [14]. Therefore, it is difficult to predict the kinetic profile of drug release from SLM or SLN.

As the lipids used in SLM do not melt at body temperature, the complete release of the active substance from the solid lipid matrix requires erosion of this matrix, for example, as a result of the action of lipolytic enzymes. Drug release from lipid microparticles is a complex process and is dependent on various formulation parameters (mainly on the distribution of the drug substance in the microparticles) and preparation conditions [18]. Physiological factors prevailing at the site of the administration of the lipid carrier are also essential for the release, such as enzymes affecting the lipid matrix, electrolytes changing the stability of the enzymes or the solubility of the drug substance, pH, ionic strength, peristalsis of the gastrointestinal tract, or the type and concentration of bile salts (in the case of oral administration) [19]. It is important to identify those factors that affect microparticles in vivo and have a real impact on the release of the drug substance from SLM, because this will facilitate the development of in vitro testing guidelines for this dosage form, which are still missing. As already justified in a previous publication [20], the release study of the active substance from lipid microparticles is a basic test both characterizing the availability of the delivered drug substance over time and correlating with the interaction of the drug substance with a carrier.

To maintain the desire effects at the target site, the efficacy of ophthalmic formulations depends not only on the drug dose in the formulation but also on the rate and mechanism of release. The conjunctival sac is a specific application site with physiological conditions such as a pH of approximately 7.4 and an osmolality of the lachrymal fluid of between 280 and 293 mOsm/kg on waking. As a result of evaporation when the eyes are open, the osmolality may vary between 231 and 446 mOsm/kg [21]. When administering eye drops, due to the relatively small volume of a single drop, the pH value of therapeutic products can vary from 3.5 to 8.5 [21].

The lack of predictability of the in vivo effects of lipid dosage forms based on the in vitro results obtained through conventional release methods results from the complexity of factors affecting the carrier in the conjunctival sac. Therefore, more studies considering the physiological factors prevailing at the site of application should be conducted to facilitate a better understanding of the pharmaceutical characteristics of lipid formulations and the interactions between lipid excipients, drug substances, and physiological environments. Therefore, release studies in biorelevant media are crucial to predict the in vivo behavior of lipid formulations. To the best of our knowledge, no studies have been conducted so far on drug release from SLM into an acceptor fluid containing physiological factors such as lysozyme enzyme, tears electrolytes and other compounds present in tear fluid. Therefore, the objective of this work was to investigate the influence of these factors on model drug solubility and release from SLM.

To date, there are only a few studies on drug release from lipid particles to have considered physiological factors by applying the biorelevant media in vitro. Research on lipid particles intended for ocular administration is practically limited to SLN. These studies were carried out using a modified Franz diffusion cell, a USP dissolution apparatus with modifications, or a modified rotating paddle dialysis bag diffusion technique, and simulated tear fluid (STF) as a release medium [22,23,24,25]. The composition of STF used in these studies was limited to 3–4 salts (usually NaCl, NaHCO_3_, CaCl_2_, KCl) and no enzymes were used. Currently, there are no data on the effect of the lysozyme enzyme on the integrity of SLM or SLN or on the release rate of the active substance from these lipid particles.

The current study focused on SLM with indomethacin (Ind) as a model drug substance. Indomethacin is a non-steroidal anti-inflammatory drug, with high analgesic and anti-inflammatory effectiveness. It is classified as BCS class II (Biopharmaceutics Classification System), so it is characterized by low solubility and good penetration through biological barriers [26]. Ind is poorly soluble in acidic media. At pH 1.1, the solubility of Ind is 0.001 mg/mL and, at pH 5.0, it is 0.01 mg/mL. Ind dissolves slightly better at a higher pH (pH 6.0: >0.5 mg/mL) [27]. The aqueous solubility does not always correlate best with the in vivo solubility and, consequently, with bioavailability. Therefore, the use of biorelevant media is a more suitable approach for mimicking the in vivo conditions in release studies. The biorelevant media are test solutions with the same physiological components and properties as the fluids they simulate, e.g., tear fluid or gastrointestinal fluid.

The aim of the conducted research was to assess the impact of physiological factors, namely components of tear fluid including lysozyme enzyme, on the structure of SLM and the release rate of the drug substance incorporated in such a carrier. The role and effect of the surfactant were also assessed.

## 2. Materials and Methods

### 2.1. Materials

Indomethacin (Ind) and urea were purchased from Fagron (Cracow, Poland), Compritol 888 ATO (glyceryl behenate) from Gattefossé (Saint-Priest, France), and Tween 80 (polysorbate 80) and citric acid from Sigma-Aldrich (St. Louis, MO, USA). Lysozyme (LS) from chicken egg white was obtained from Pol-Aura (Olsztyn, Poland). Sodium chloride, potassium chloride, ammonium chloride, magnesium chloride, and anhydrous calcium chloride were sourced from POCH (Gliwice, Poland), sodium bicarbonate from Chempur (Piekary Śląskie, Poland), and lactic acid 80% from B&K (Bytom, Poland). All other chemicals used were of analytical reagent grade. A Milli-Q system (Millipore, Milford, MA, USA) was employed for obtaining high-quality water.

### 2.2. Preparation of SLM Dispersions

A hot emulsification technique was used to prepare SLM dispersions. The lipophilic phase (lipid—Compritol 10% *w*/*w* with dissolved drug substance—Ind 0.2% *w*/*w*) and hydrophilic phase (water and surfactant—Tween 3.0%) were separately heated to the temperature of 80 °C in a water bath and then the combined phases were mixed for 5 min (8000 rpm) using an Ultra-Turrax high-shear dispersing mixer (T25 Janke-Kunkel, IKA Labortechnik, Staufen, Germany). The obtained emulsion was finally cooled in an ice bath to solidify lipid microparticles. The resulting SLM dispersions were stored in a refrigerator. The entire procedure has been previously described [2]. The *placebo* SLM dispersion was prepared in the same way, without the drug substance.

### 2.3. Characteristics of SLM Dispersions

The obtained SLM formulations were characterized in terms of particle size, drug substance distribution, and microparticles morphology, as already described in previous reports [2,14,28].

The particle size distribution in SLM dispersions was measured by the laser diffraction method with a Universal Liquid Module (Beckman-Coulter LS 13 320, Indianapolis, IN, USA). The obtained results were presented as d_10_, d_50_, and d_90_ values, which are the measures of the maximum diameters of 10%, 50%, and 90% of the detected particles, respectively [28].

The distribution of Ind between the individual phases of the SLM dispersion was determined as follows [14]. The fraction of free drug dissolved in the aqueous phase was determined after ultrafiltration. The sum of the Ind amount located in the aqueous phase and at the surface of the microparticles was determined in the supernatant, after dilution of the SLM dispersion with methanol, vortexing, and centrifugation. The amount of the Ind localized in the interphase was calculated by subtracting the quantity found in the aqueous phase from this value. The remaining amount was considered the drug fraction incorporated in the lipid matrix [14].

Surface morphology examination of the tested microparticles was carried out using a scanning electron microscope (SEM) Phenom Pro (Phenom World Thermo Fisher, Eindhoven, The Netherlands). An SLM sample was placed on the carbon adhesive tape, and after water evaporation under ambient conditions, the sample was coated with a thin layer of gold [28]. An acceleration voltage of 15 kV was applied to record micrographs at magnifications of 5000, 10,000, and ×20,000. SLM formulations were also observed using an optical microscope (Nikon Eclipse 50i, Nikon Corporation, Tokyo, Japan).

### 2.4. Solubility of Ind in Acceptor Media

The solubility of Ind was tested in the fluids considered for use as acceptor media in the release study. These were (i) artificial tear fluid (AT), (ii) 0.9% sodium chloride solution (NaCl), (iii) 5% polysorbate 80 solution (Tw), and their mixtures with the addition of lysozyme (LS 1.4 mg/mL). Three-component mixtures were also examined, i.e., artificial tear fluid or sodium chloride solution with the addition of both polysorbate and lysozyme. A summary of all tested solutions is provided in Section 3.2. The composition of the artificial tear fluid based on the available data [29,30,31] was as follows: sodium chloride (99 mmol/L), potassium chloride (20 mmol/L), ammonium chloride (3 mmol/L), magnesium chloride × 6H_2_O (0.8 mmol/L), anhydrous calcium chloride (0.8 mmol/L), sodium bicarbonate (28 mmol/L), lactic acid (3.4 mmol/L), citric acid (0.04 mmol/L), and urea (5.5 mmol/L). The osmotic pressure of the acceptor fluids was determined by the freezing-point method using Microosmometer Digital (Knauer, Berlin, Germany).

An excess of Ind was mixed with the tested solvents using a magnetic stirrer with a rotation speed of 300/min at 30 ± 1 °C in test tubes. After 24 h, the final suspensions were centrifuged (3500 rpm for 5 min) and filtered through a cellulose acetate membrane filter (Alchem, Torun, Poland) with a pore size of 0.2 µm. The concentration of Ind was determined by high-performance liquid chromatography (HPLC, see below), after appropriate dilution with methanol. The solubility test was carried out in triplicate.

### 2.5. Drug Release Study

The release study was conducted using the dialysis bag method, with the fluids presented in Section 2.4. The volume and type of acceptor fluid were selected to ensure *sink* conditions.

A dialysis tubing cellulose membrane (flat width 25 mm) with a molecular weight cut-off (MWCO) of 14 KDa (Sigma-Aldrich, St. Louis, MO, USA) was prepared prior to this study, as recommended by the manufacturer, to use in the dialysis bag diffusion technique. Five milliliters of SLM dispersion was placed in a dialysis bag. For some experiments, the dispersion in the dialysis bag was diluted (in a 4 + 1 ratio) with the solution used as an acceptor fluid (usually with dissolved lysozyme). This procedure allowed the lysozyme to be introduced directly into the bag. The dialysis bag was placed in a glass beaker with 70.0 mL of acceptor medium and incubated at 37 °C in a mechanical shaking bath (150 cycles/min). At the predetermined time points, 1.0 mL of the acceptor fluid was withdrawn and replaced with fresh fluid. The concentration of Ind in the acceptor fluid was determined by HPLC after appropriate dilution with methanol. The cumulative amount of the released drug was expressed as a percentage of the theoretical drug content. The residue from the dialysis bag after the release test was observed under an optical microscope and was also subjected to particle size measurement by laser diffraction (as described in Section 2.3).

### 2.6. Assessment of SLM after Incubation with Natural Tears

SLM dispersions (*placebo* and drug-loaded) were incubated with natural tear fluid (1 + 1) at 37 °C. SLM with Ind and *placebo* SLM were studied in the same way. Tear fluid was collected with a sterile glass pipette from the medial angle of the eye of an author of this publication. After 5 days of incubation, the particles’ shape and morphology and the agglomeration of the microparticles were assessed microscopically.

### 2.7. Analysis of Ind Concentration by HPLC

The concentration of Ind in the tested samples was analyzed by reverse-phase high-performance liquid chromatography (RP-HPLC) using Prominence LC-2030C 3D apparatus (Shimadzu Corporation, Kioto, Japan) [20]. A 125 × 4 mm column (LiChrospher 100 RP-18, Merck KGaA, Darmstadt, Germany) was used as the solid phase. The mobile phase consisted of acetate buffer and methanol at a ratio of 35:65 (*v*/*v*). The flow rate was 1 mL/min, the temperature was 30 °C, and the detection wavelength of the UV detector was 215 nm. A sample volume of 20 µL was delivered using an autosampler.

### 2.8. Statistical Analysis

The obtained data were statistically analyzed using a one-way analysis of variance (ANOVA). A *p*-value < 0.05 was considered to be significantly different.

## 3. Results

### 3.1. Characterization of SLM Dispersions

The sizes of the tested lipid microparticles, as measured by laser diffraction, are presented in Table 1, which also presents the distributions of Ind in different phases of SLM dispersion as percentages of the total drug content [14]. In the tested SLM-Ind formulation, less than 1.5% of the drug substance was localized in the aqueous phase, while a large fraction (almost 80%) was found in the interphase (on the surface of the lipid particles). The remaining part of the drug substance (approximately 20%) was identified as incorporated in the lipid matrix. We prepared SLM with sizes ranging from one to several micrometers (Table 1), which are suitable for ocular administration, because this size is considered to be non-irritating after administration to the eye [32]. The tested lipid microparticle dispersions were also characterized by the desired long-term stability, which was described in previous studies [14,33].

### 3.2. Solubility Studies and Properties of Acceptor Media

**Figure 1:**
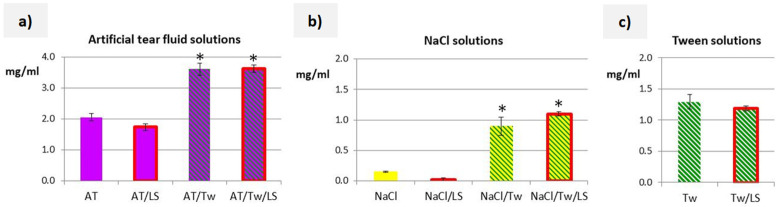
The solubility of Ind (mean ± SD, * statistically significant difference) in acceptor media: (**a**) in artificial tear fluid, (**b**) in 0.9% sodium chloride solution, and (**c**) in 5% Tween 80 solution. Solutions with the addition of lysozyme are marked with a red border.

When comparing artificial tear fluid, 5% Tween, and 0.9% sodium chloride solution, the highest Ind solubility was obtained in artificial tear fluid (2.05 mg/mL), which was 1.5 times higher than in Tween solution (1.29 mg/mL). The lowest solubility of Ind (0.15 mg/mL) was determined in sodium chloride solution. The addition of Tween (5%) increased the solubility of Ind by almost twice in artificial tear fluid (3.6 mg/mL), while in the mixture of Tween and sodium chloride solution, the solubility was six times higher (0.89 mg/mL) than in sodium chloride solution. The addition of lysozyme (1.4 mg/mL) had no effect on the solubility of Ind, regardless of the solution’s composition (Figure 1).

The tested acceptor media were generally isoosmotic, except for the highly hypotonic polysorbate solutions (Table 2). The pH values ranged from 8 to 4.5, with the highest pH of artificial tear fluid and lowest pH demonstrated by solutions of sodium chloride and Tween with lysozyme (NaCl/LS and Tw/LS). Generally, it was observed that the addition of lysozyme to sodium chloride solution and/or Tween solutions resulted in a pH decrease of from 0.9 to 1.6 units.

### 3.3. Drug Release Study

**Figure 2:**
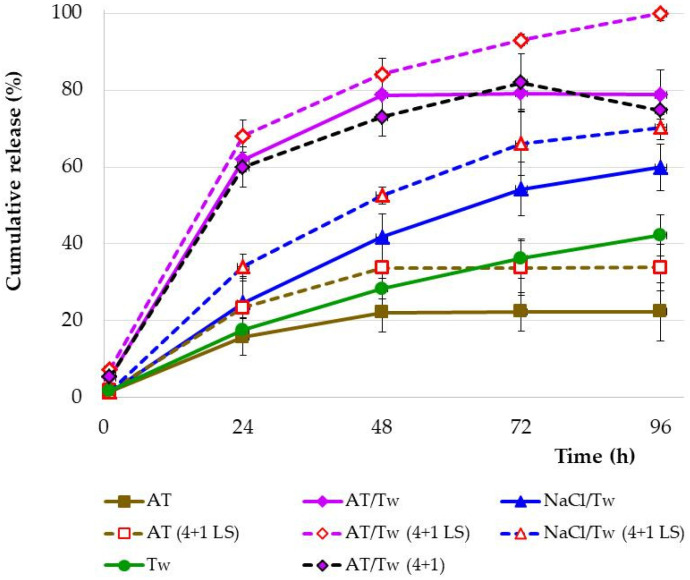
Release profiles of Ind from SLM dispersions (n = 3–5), with or without dilution (4 + 1) of the dispersions in the dialysis bag, into various acceptor fluids. The solid line indicates SLM dispersions tested without dilution, the dashed line indicates SLM dispersions diluted (4 + 1) in the dialysis bag, and the profiles obtained in the presence of lysozyme are marked with red-colored dots.

When comparing the Ind release profiles from the undiluted lipid microparticle dispersions into four different acceptor fluids, significant differences were found, as presented in Figure 2. During the study (96 h) of undiluted SLM dispersions without the addition of lysozyme, 100% release of the drug substance was not achieved, although all acceptor fluids provided *sink* conditions. Unlike the Tween and NaCl/Tw medium, a *plateau* effect was observed after 48 h of the study in the other two acceptor fluids, but at a different level. Only 22% of Ind was released into artificial tear fluid and almost 79% into AT/Tw. In the study with polysorbate solution with or without sodium chloride, a continuous increase in Ind was observed in the acceptor fluid, resulting in the release of 60% (NaCl/Tw) or 42% (Tw) of Ind during the 96 h of the study.

In the next step, the release of Ind from the lipid microparticles was studied in the presence of the lysozyme enzyme (Figure 2). For this purpose, the release of Ind from a dispersion of microparticles diluted (4 + 1) in a dialysis bag was studied. This procedure allowed the lysozyme enzyme to be introduced into the bag for direct contact with the microparticles. The lysozyme concentration was the same in all tested systems (1.4 mg/mL). Each time, release studies resulted in an increase in the amount of released Ind in the presence of lysozyme, regardless of the type of acceptor fluid. However, only in the simultaneous presence of artificial tear fluid and polysorbate (AT/Tw 4 + 1 LS) was 100% release of the drug substance achieved.

The addition of lysozyme to tear fluid (AT 4 + 1 LS) increased the release of Ind in the first stage of the study (48 h), from 22% to 34%, without changing the overall release profile (*plateau* effect from 48 h). The addition of lysozyme to tear fluid in the presence of polysorbate (AT/Tw 4 + 1 LS) increased the amount of released Ind by up to 100% at 96 h of the study (lysozyme inside the dialysis bag). Sodium chloride solution was also used to study the release of Ind, although due to the previously determined solubility of Ind (Figure 1b), it was always tested with polysorbate (NaCl/Tw). The introduction of lysozyme into this experiment (NaCl/Tw 4 + 1 LS) resulted in the release of 70% of Ind, compared to 60% in the experiment without the enzyme (NaCl/Tw).

There was also an experiment performed (AT/Tw 4 + 1) where the dispersion in the bag was diluted with artificial tear fluid without the presence of lysozyme. The results obtained were almost identical to the model without lysozyme and without SLM dilution in the bag (AT/Tw).

### 3.4. Assessment of SLM from the Dialysis Bag after the Release Study

**Figure 3:**
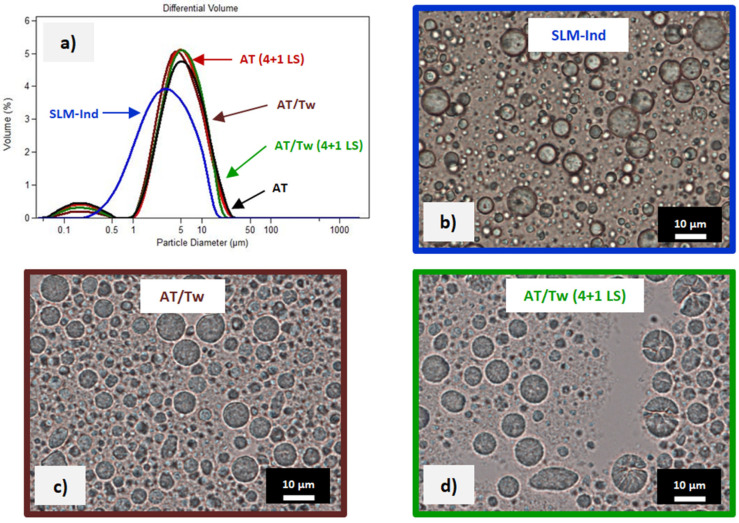
(**a**) Size distribution of microparticles before and after release test into tear fluid or tear fluid with polysorbate. Microscopic images of (**b**) Ind-loaded SLM after preparation and (**c**,**d**) the residue from the bag after the release study into selected acceptor media.

The particle size distribution measured in the release test residue collected from the dialysis bag is shown in Figure 3. The obtained results were compared with the size distribution determined in the freshly prepared dispersion of lipid microparticles with Ind.

After the 96 h of the release study, the average size of the microparticles had increased from approximately 3.8 μm to approximately 4–5 μm (Figure 3a). At the same time, the size distribution became bimodal, presenting an additional slight fraction of small particles below 1 µm (smaller than those present in the dispersion before testing). A similar effect occurred both in artificial tear fluid and AT/Tw.

As described in Section 4.4, the location of a significant fraction of Ind is superficial, so even partial dissolution and removal (e.g., during release) will result in a decrease in particle size compared to those recorded in the de novo formulation. This effect will be more notable for small particles that have a different surface-area-to-volume ratio. Generally, drug release is faster from smaller particles compared to bigger ones, which is a behavior observed both for polymeric and lipid microparticles [3].

During microscopic observations of the residues after the release test (Figure 3b–d), single spherical microparticles were still clearly visible, although some agglomerates of smaller particles and very fine particles difficult to distinguish in the microscopic image were also observed. However, the main difference was observed in the dispersion samples diluted with medium containing the enzyme (AT/Tw 4 + 1 LS). These were single microparticles with cracks and structural deformations (Figure 3d). Such microparticles were not reflected in the particle size distributions measured by laser diffraction (Figure 3a), although it can be assumed that deformed lipid microparticles in a laser diffractometer may be mistakenly measured as larger particles. This is due to the measurement principle on which laser diffraction is based. Particle shape can have a profound impact on particle size distribution measurements because an equivalent spherical diameter is usually determined by measuring a size-dependent property of a particle and relating it to the diameter of a sphere. For the same reason, the above measurement method does not distinguish between individual particles and their agglomerates. Therefore, the obtained results should always be verified and considered in relation to microscopic observations.

Taking into account all the above-mentioned factors helps explain the observed bimodal particle size distribution, which was also confirmed by microscopic observations and further studies with natural tear fluid.

### 3.5. Assessment of SLM after Incubation with Natural Tears

**Figure 4:**
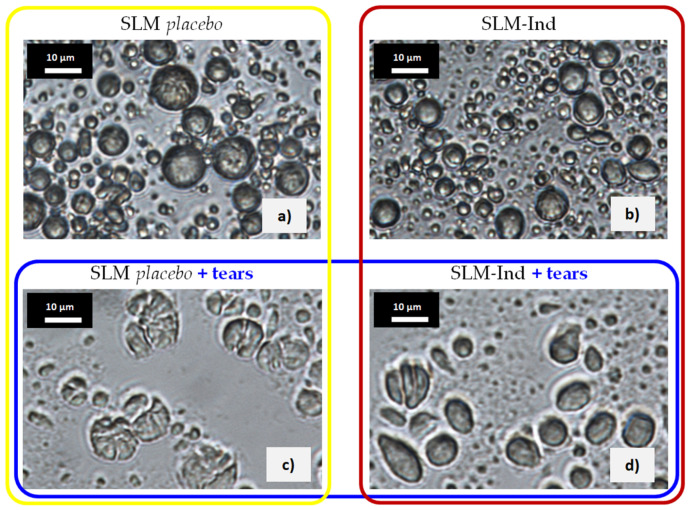
Optical microscope images of (**a**) nontreated SLM *placebo*; (**b**) nontreated Ind-loaded SLM; (**c**) SLM *placebo* after incubation with natural tears; and (**d**) Ind-loaded SLM after incubation with natural tears. *Placebo* SLM are marked in yellow, Ind-loaded SLM are marked in red, and SLM (*placebo* or Ind-loaded) after incubation with natural tears are marked in blue.

After the incubation of the *placebo* lipid microparticles with tear fluid, numerous deformed particles, or ones with visible cracks, were found in the microscopic image (Figure 4a vs. Figure 4c). The smallest particles were less numerous, while a uniform “lipid” background with particles difficult to distinguish was observed. Similar changes were observed in microparticles with Ind, although there were significantly fewer cracked and deformed particles in these images (Figure 4d).

**Figure 5:**
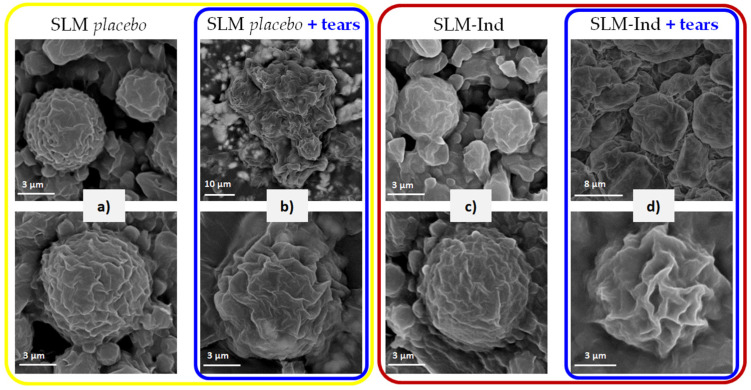
SEM images of (**a**) nontreated SLM *placebo*; (**b**) SLM *placebo* after incubation with natural tears; (**c**) nontreated Ind-loaded SLM; (**d**) Ind-loaded SLM after incubation with natural tears. *Placebo* SLM are marked in yellow, Ind-loaded SLM are marked in red, and SLM after incubation with natural tears are marked in blue.

SEM images of the same microparticles confirmed the observations from the optical microscope. After contact with tear fluid, particle aggregates were visible in the *placebo* SLM (Figure 5b), presumably originating from the fragments of deformed microparticles visible in wet images (photos of dispersion from an optical microscope). A different shape of the microparticles after incubation was also visible (Figure 5b,d vs. Figure 5a,c). When comparing the appearance of individual particles, the main surface differences were the much greater porosity of SLM exposed to tears, as well as the effect of “blurring” the shape of the particles.

## 4. Discussion

The described methods of analysis are a continuation of studies on the release of drug substances from SLM [20]. Previous studies have already demonstrated the role of the acceptor fluid and the importance of the choice of release testing method for the results obtained.

Since the developed formulations have shown potential as promising dosage forms for topical administration to the eye, this study focused primarily on the in vitro effect of artificial tear fluid and the lysozyme enzyme on lipid microparticles and, consequently, the release profiles.

The release of a drug is important for its bioavailability and therapeutic effectiveness [34]. A properly designed in vitro release study allows not only to assess the quality and stability of the medicinal product but also provides the basis for predicting the in vivo behavior. Moreover, conducting a study in accordance with pharmacopoeial guidelines and the recommendations of registration organizations allows for the comparison of study results between centers. It is critical for procedures to be standardized, which helps to show consistent quality in production and may serve as a predictive measure of efficacy [34].

### 4.1. Solubility Studies

An important factor in release studies is the solubility of the drug substance in the acceptor fluid. Satisfactory, although not the highest, solubility of Ind was obtained in Tween solution. The solubility of Ind in artificial tear fluid was higher than in the Tween (Figure 1a,c), which can be explained by the composition of the artificial tear fluid. Sodium citrate and urea, thanks to enhancing the solvation of the drug molecules by water [35], are, among others, responsible for the observed effect. The addition of polysorbate further increased the solubility of Ind in artificial tear fluid and sodium chloride solution, without significantly affecting the pH values of these liquids (pH change of no more than 0.4 units). On the other hand, the addition of lysozyme to any liquid did not significantly affect the solubility of the tested substance, although in unbuffered systems, a decrease in pH ranging from 0.9 to 1.6 units was visible (Table 2). After adding lysozyme to artificial tear fluid, an increase in pH of approximately 0.5 units was recorded. There were no significant differences in solubility in the presence of lysozyme, although such a correlation would be expected due to the change in pH. In summary, an increased solubility of Ind cannot be expected with an increasing pH. In the conducted studies, this relationship was very weakly visible, and differences in pH did not explain the differences in the solubility of the active substance.

Except sodium chloride solution without the addition of polysorbate, the results of the solubility test allow for the use of all liquids in accordance with the *sink* principle as acceptor media in the proposed research model (maximum 10 mg of Ind in the dialysis bag).

As already explained in the introduction, the use of the so-called biorelevant media, i.e., in our case, artificial tear fluid, is an appropriate approach to imitate in vivo ophthalmic conditions. The artificial tear fluid composition and lysozyme concentration were selected based on available data [29,30,31]. So far, there have been only two SLM studies using biorelevant media, but in respect to oral rather than ocular delivery [36,37].

### 4.2. Drug Release Studies

At the present time, there is no harmonized regulatory standard (FDA/EMA) for in vitro dissolution/release methods applicable to the assessment of multi-compartment lipid carriers, e.g., SLM, especially when they are intended for administration to the eye. There is a paucity of information in this critical field. To the best of our knowledge, no studies on lipid microparticles are yet available to assess the effect of lysozyme enzyme on the lipid matrix of microparticles, and thus, on the release process of the incorporated active substance. Therefore, it is desirable to develop appropriate in vitro testing methods to improve development and screening tests, ensure the quality and safety of the developed formulations, and, in the future, advance the commercialization activities of SLM ocular dispersions.

Among all the in vitro tests for microparticles, the release study is one of the key experiments. The in vitro release kinetics of SLM provide critical information regarding their ability to modify drug release, which is an important parameter to be considered in the assessment of the efficacy and quality of products. If a release study is planned and performed correctly, the obtained results can be related not only to the distribution of the active substance in microparticles but, above all, to the in vivo behavior of the dosage form.

In studies conducted with artificial tear fluid, less than 50% of Ind was released. Although the addition of lysozyme increased the release by up to twofold within 48 h, ultimately, no more than 41% of the dose was released from SLM. Although all tested solutions guarantee *sink* conditions, significantly less Ind was released to artificial tear fluid (surfactant-free medium) than would be expected.

Regardless of the acceptor medium, a *sink* condition was used, under which the volume of the release medium should be three times larger than that required to obtain a saturated drug solution [38]. Hence, the solubilities of model drugs in all considered acceptor media were determined prior to the release study.

Because of its weakly acidic nature (pKa of 4.5), Ind presents a very low aqueous solubility that slightly increases when changing from an acidic to a neutral/alkaline pH [39]. The use of polysorbate (Tw) as a surface active substance in the composition of artificial tear fluid is justified by the position of the European Medicines Agency. According to EMA guidelines [40], in general, an aqueous environment should be used. To obtain adequate release of active substances that are poorly soluble in water under such conditions, the use of surfactants is justified [40]. Addition of polysorbate resulted in a statistically significant increase in solubility and in the release of Ind from SLM (even 2-fold in AT/Tw vs. AT, Figure 2). However, a *plateau* effect was still observed, and the drug substance release level did not exceed 79%. First, solubility (which increased in the presence of Tween) can be considered the driving force for the release of Ind from SLM, even though both artificial tear fluid and AT/Tw fluid provided *sink* conditions and did not differ in terms of pH.

As already described by Siepmann et al. [38], limited drug solubility effects can play a major role in the control of drug release. In vivo, the released drug might be rapidly transported away from the conjunctival sac, e.g., due to absorption into the blood stream or nasolacrimal drainage. The in vitro experimental model used is “a closed system” and eventually drug saturation effects in the surrounding release medium might artificially occur, “falsifying” the resulting release kinetics [38]. To avoid such errors, *sink* conditions are provided. However, despite this, the dissolution rate is proportional to the concentration gradient, according to the Noyes–Whitney equation [41]. In addition to the surface effect, this is also one of the effects influencing the better release of Ind in the presence of polysorbate. In this way, the addition of Tween in vitro goes some way to counterbalance the described in vivo effects that cannot be mimicked in a closed release study model.

Among other substances ensuring *sink* conditions and the release of the drug substance, propylene glycol with PBS (40:60) was used in studies on the release of loratadine from SLM intended for nasal administration [7]. The 5% concentration of polysorbate we used was negligible compared to the content of propylene glycol in the above-mentioned acceptor fluid. Similarly, release studies of zidovudine were performed in a water–methanol mixture (70:30, *v*/*v*) [8]. Employing methanol as a cosolvent was necessary to increase the low solubility of the drug in water, thus ensuring *sink* conditions. The same tests were also carried out in the presence of 0.5% Tween 20, confirming its importance for the release of the drug from SLM. The drug was released in larger quantities, although the addition of Tween did not influence the initial portion of the release curves, which were characterized by a better effect of prolonged release [8].

As the microscopic images showed, the surface of the microparticles is not smooth, but irregular and wrinkled. By interacting with this surface, polysorbate causes its further increase in size by deepening the folds and grooves due to the dissolution and release of Ind from SLM. This will have implications for the performance of the other components of the acceptor fluid. Moreover, according to previous experience [14,20], the release of no more than 79% of Ind within 96 h, despite the presence of polysorbate, was associated with the distribution of the drug substance in various phases of SLM dispersion. Therefore, it can be concluded that the surfactant is important for the release of Ind primarily located on the surface. This phenomenon may be related to solubilization and dissolution, and the transfer of Ind from the surface of the particles to the acceptor fluid, without affecting the remaining (incorporated in the lipid) fraction of the drug. The importance of surfactants in release studies was also pointed out by Bertoni et al. [3], especially in relation to smaller lipid microparticles—such as those studied by us, rather than larger ones (>250 μm).

Due to the lipid nature of the microparticles, in which at least part of the drug substance is incorporated in the particle matrix (Table 1), the release of the active substance occurs not only because of diffusion but also as a result of digestion and enzymatic degradation of the lipid matrix by physiological enzymes, depending on the route of administration [3].

Incomplete release and reaching a *plateau* effect after 48 h, at a release level of 79% (AT/Tw), confirms the incorporation of approximately 20% of Ind into the SLM lipid matrix (Table 1). If the lipid microparticles do not dissolve/melt in the aqueous environment and are not treated to enzymatic degradation, part of the drug substance enclosed/incorporated in the microparticles cannot be released. Therefore, taking into account the route of administration of the tested SLM with Ind to the eye, the use of the enzyme lysozyme, which occurs under physiological conditions in tears, was considered important in the next stage of release studies. Lysozyme is abundant in the tear fluid and has antimicrobial activity. Although lysozyme is not a typical lipolytic enzyme digesting the lipid matrix of microparticles, the interaction of lysozyme present in the aqueous layer of the tear film with lipids (fatty acids, cholesterol, phospholipids, and their mixtures) present in the lipid layer and other lipid films is a subject of current research [42]. Therefore, it was considered justified to clarify whether lysozyme used in vitro mimics the in vivo conditions.

In the real situation, when the SLM dispersion was administered in the conjunctival sac, the lipid microparticles mixed with the tear fluid present on the surface of the eye in which lysozyme was present; so, the use of a release medium with the addition of the lysozyme enzyme is more suitable to mimic the in vivo conditions than fluids without the enzyme.

Lysozyme is a hydrolytic enzyme composed of 129 amino acids, and apart from its presence in tears, it is also present in body fluids such as saliva, gastric juice, and airway mucus secretions. The use of the lysozyme enzyme in research is difficult because its activity is a function of both pH and ionic strength [43]. Although lysozyme is active in a relatively broad pH range from 6.0 to 9.0 (some sources indicate pH 5.0–8.0 as optimal for lysozyme activity), it should be remembered that at pH 6.2, the maximum activity is observed over a wider range of ionic strengths (0.02–0.100 M) than at pH 9.2 (0.01–0.06 M).

The addition of lysozyme to the dialysis bag, in a release study conducted into NaCl/Tw fluid, resulted in an increase in the amount of released substance by approximately 10%. The same effect was observed in artificial tear fluid. Although the pH of the sodium chloride solution fluids was the lowest, the release into NaCl/Tw with or without lysozyme was similar and not the slowest compared to artificial tear fluid. In release tests conducted with AT/Tw, the use of lysozyme increased the percentage of the released dose by up to 100%.

The influence of lysozyme was noted regardless of the type of acceptor fluid. However, the lack of significant changes in the solubility of Ind in the acceptor fluid with the addition of lysozyme (Figure 1) indicates another mechanism of increased release, directly related to SLM. The changes caused by lysozyme in the release profiles depended on its effect on lipid microparticles, as well as on the type of acceptor fluid into which the release took place (including the addition of polysorbate). The release of 100% Ind within 96 h was noted only in the AT/Tw (4 + 1 LS) experiment. This means that the entire dose of the substance contained in SLM is released, regardless of the degree of incorporation into the microparticles and distribution in the individual phases of the dispersion. Consequently, it should be considered whether lysozyme affects the microparticles strongly enough to release even that part of the substance that is incorporated in the lipid matrix of the particles, or whether the incorporation of Ind in glycerol behenate microparticles occurs only in the outer lipid layers of the microparticles, meaning that Ind is relatively easily accessible. An attempt to explain the above effect was made in further studies that assessed the content of the dialysis bag after the release test, as well as SLM after incubation with natural tear fluid (see description in Section 4.3 and Section 4.4). The results of the release studies and the assessment of the residue after the test clearly indicated that it is possible to release 100% of Ind from microparticles without a complete degradation of the lipid matrix.

Diluting SLM in the bag mimics direct mixing of SLM with tear fluid during ocular application. When analyzing the obtained results, it was found that despite diluting the SLM dispersion in the dialysis bag with artificial tear fluid and polysorbate, without lysozyme (AT/Tw 4 + 1), 100% of Ind was not released (Figure 2). Similarly, complete release was not achieved without the presence of Tween (AT 4 + 1 LS). The comparison of the effect of lysozyme on the release of Ind from SLM in the presence of AT/Tw or NaCl/Tw confirmed that the release of Ind delivered in the form of SLM to the ocular surface will depend not only on the presence of lysozyme but will also be related to the tear fluid in the conjunctival sac (components, pH, ionic strength). Thus, only dilution of the microparticles with acceptor fluid and the addition of lysozyme (AT/Tw 4 + 1 LS), thanks to the simultaneous presence of lysozyme, Tween, and artificial tear fluid, resulted in the release of the entire dose of the drug substance (Figure 2).

The complete release of the active substance suggested the localization of Ind at the surface and near the surface in the outer lipid layers of the SLM. Despite the lack of interaction with the core of the microparticles, Tween and lysozyme turned out to be important components of the acceptor fluid. Their slow and long-lasting action resulted in the release of an Ind fraction, which was not dissolved during the determination of the fraction located in the interphase (Ind distribution test, Section 2.3) even by methanol. Artificial tear fluid was also found to be an essential component of the acceptor fluid, without which complete release could not be achieved. The superficial changes observed in SLM incubated with tear fluid (Figure 5) confirmed the role and the in vivo importance of all tear fluid components. It was clear that no polysorbate would be present in vivo after application to the eye. However, the processes occurring on the surface of the microparticles were sufficient to completely release Ind in vivo, and the same processes were observed after incubation of SLM with tears without polysorbate. Moreover, in the biological system (in the eye), the released drug substance continuously undergoes further transformations (absorption, dilution with tear fluid, elimination), which will lower its concentration and promote further release.

It is also worth noting that the burst effect, which was reported in relation to the release method without a dialysis membrane [20], was not observed in the method with a dialysis bag. Very rapid release of a significant fraction of Ind from SLM did not occur in the initial phase of this study, regardless of the test conditions. These results confirmed the prolonged release effect, which may be difficult to achieve. For example, Gugu et al. [4] studied aspirin-loaded SLM. Their microparticles in an in vitro release study presented a very high drug release within the first 0.5–1 h, so an effect like a burst release.

### 4.3. Assessment of SLM from the Dialysis Bag after the Release Study

The analysis of the residues in the dialysis bag after testing the release of Ind from SLM did not indicate any influence of the type of acceptor fluid (including the presence of polysorbate) on the particle size measured by laser diffraction (Figure 3a). Very similar distributions were obtained even in the presence of the lysozyme enzyme. In all measurements, a peak of particles smaller than those previously detected in the dispersion was visible, which indicated partial degradation and spontaneous hydrolytic decomposition in an acidic or alkaline environment, especially the smallest particles in the dispersion. At the same time, the agglomerates observed in the microscopic image formed by, among others, partially degraded particles, may have explained the shift of the main peak in the particle size distribution toward larger particles. In general, however, spherical microparticles were still visible after the release test (Figure 3b–d).

### 4.4. Assessment of SLM after Incubation with Natural Tears

Finally, in order to confirm or deny the influence of the lysozyme enzyme as well as the physiological conditions in the eye (electrolytes present in tears, pH) on the release of Ind from lipid microparticles, the SLM dispersions were incubated with natural tears. The changes observed in SLM with Ind were very similar to those after the bag residue study, and even more pronounced and intensified when *placebo* SLM were incubated with natural tears (Figure 4c,d). This may have resulted from the previously discussed distribution of Ind (Table 1), which was largely located on the surface of the microparticles in the so-called interphase, also co-created by a surfactant stabilizing the suspension of SLM. Unlike *placebo* microparticles, this hindered direct access of lysozyme to the lipid matrix in Ind-loaded SLM. Moreover, in the SLM *placebo*, aggregates of particles with a clearly disturbed individual structure were visible in the SEM images (Figure 5b). The SEM observations were also confirmed by optical microscopy, which revealed further differences between microparticles. In the *placebo* formulations, many particles were deformed and appeared to be cracked and disintegrating (Figure 4c). The greater intensity of changes visible in *placebo* SLM was consistent with previous observations. Although the SEM images failed to visualize the characteristic disintegrating microparticles from the optical microscope, which may have resulted from the observation conditions (dry microparticles and wet dispersion), differences in the shapes of the particles surfaces were visible. Our comparison of *placebo* and Ind-loaded SLM before (Figure 5a,c) and after (Figure 5b,d) incubation with tears clearly indicated greater wrinkles, sharp edges, and a simultaneous blurring effect of the entire particles compared to the smoother and more spherical microparticles with soft edges before incubation.

To summarize, it should be stated that the effect of lysozyme on lipid microparticles was confirmed, although this effect does not result from complete degradation of the lipid matrix by the enzyme. Structural changes observed in microparticles incubated with tears proved the impact of physiological components on SLM. At the same time, sodium chloride solution alone in the presence of lysozyme was insufficient to obtain effects similar to those of natural tears in the in vitro release test, suggesting an influence of tear fluid (components, pH, ionic strength) on enzyme activity and, consequently, an influence on SLM. This confirms the need for the simultaneous use of all physiological factors occurring in vivo, in combination with a surfactant that guarantees the dissolution of a poorly soluble active substance. Obtaining the release of the total dose of Ind without simultaneous degradation of the microparticle structure also suggests the localization of the active substance on the surface and in the outer layers of the lipid matrix of the microparticles, making it relatively easily available for release, without the complete degradation of the lipid matrix. Despite this fact, in the studied models without tear fluid or lysozyme, the release of Ind did not exceed 60% within 96 h.

## 5. Conclusions

In the conducted studies, there was no relationship between the solubility of Ind and the pH of the tested acceptor fluids. The *sink* conditions of the selected acceptor media depended on their composition. Therefore, in release studies, there was no correlation between the amount of substance released and pH.

The use of artificial tear fluid as a biorelevant medium in the study of Ind release was considered justified. The release of Ind from SLM also depended on lysozyme and Tween, which, when used alone, increased the release; but only their combined use with artificial tear fluid resulted in the complete release of the drug substance from the microparticles. This was the result of the distribution of Ind in the SLM (mainly on the surface and in the outer layers of the microparticles). Therefore, the release of 100% Ind without degradation of the lipid matrix of the microparticles (which was confirmed by post-release residue studies) was solely due to surface changes occurring under the influence of lysozyme, Tween, and components of artificial tear fluid. These results were also confirmed by studies conducted after the incubation of SLM with tear fluid, which revealed the same structural changes under the influence of physiological factors.

The comparison of the effect of tears on SLM-Ind with the above results allows to expect a similar behavior of lipid microparticles in vivo after application to the conjunctival sac.

The conducted research provides fundamental insights into the importance and influence of the individual factors on the in vitro release process, such as the action of lysozyme enzyme, ionic strength, ionic composition (osmotic pressure at the physiological level provided only by the addition of sodium chloride, or the presence of various electrolytes and organic compounds physiologically present in the tear fluid), solubility of the active substance, as well as the presence of surfactant. The obtained results elucidate the critical variables for the development of the in vitro release method from SLM dispersions. It is expected that release studies using physiological factors acting in the conjunctival sac on SLM will provide, at an early stage of formulation development, sufficient guidance on the properties of the dosage form, and will facilitate their modification and design in such a way as to achieve the desired effect, without the need to conduct experiments in subsequent phases (ex vivo or in vivo on animals) before the final and desirable composition of SLM dispersion can be developed. The optimization of the release process based on the indicated parameters will facilitate development research and might also support the regulatory assessment of this dosage form, and thus the marketing authorization of a multi-compartment carrier in the form of lipid microparticles.

## Figures and Tables

**Table 1:** Characteristics of tested SLM dispersions (mean ± SD%, n = 3).

Formulation	Particle Size (µm)			Ind Distribution (%)		
	d_0.1_	d_0.5_	d_0.9_	Aqueous Phase	Interphase	Lipid Matrix
SLM *placebo*	0.77 ± 0.16	2.13 ± 0.08	5.95 ± 0.21	–	–	–
SLM-Ind	0.87 ± 0.25	2.87 ± 0.14	8.39 ± 0.43	1.2 ± 18	78.2 ± 5.2	20.6 ± 4.8

**Table 2:** Characteristics of the investigated acceptor media: pH and osmotic pressure (mean ± SD%, n = 3), and their composition (*w*/*w*%).

Acceptor Media	Tw	LS	NaCl	AT	Water	pH	Osmotic Pressure [mOsm/kg]
AT	–	–	–	100.0	–	7.41 ± 0.07	307 ± 6
AT/LS	–	0.14	–	to 100.0	–	7.86 ± 0.11	318 ± 4
AT/Tw	5.0	–	–	to 100.0	–	7.52 ± 0.09	338 ± 5
AT/Tw/LS	5.0	0.14	–	to 100.0	–	7.99 ± 0.07	343 ± 7
NaCl	–	–	0.9	–	to 100.0	6.14 ± 0.01	311 ± 2
NaCl/LS	–	0.14	0.9	–	to 100.0	4.53 ± 0.12	319 ± 4
NaCl/Tw	5.0	–	0.9	–	to 100.0	5.74 ± 0.05	329 ± 3
NaCl/Tw/LS	5.0	0.14	0.9	–	to 100.0	4.85 ± 0.18	340 ± 8
Tw	5.0	–	–	–	to 100.0	6.15 ± 0.11	15 ± 7
Tw/LS	5.0	0.14	–	–	to 100.0	4.59 ± 0.16	24 ± 4

Tw: Tween 80, LS: lysozyme, NaCl: sodium chloride, AT: artificial tear.

## Data Availability

Data are contained within the article.
